# A Late Pleistocene hominin footprint site on the North African coast of Morocco

**DOI:** 10.1038/s41598-024-52344-5

**Published:** 2024-01-23

**Authors:** Mouncef Sedrati, Juan A. Morales, Jérémy Duveau, Abdelmounim El M’rini, Eduardo Mayoral, Ignacio Díaz‐Martínez, Edward J. Anthony, Glen Bulot, Anass Sedrati, Romain Le Gall, Ana Santos, Jorge Rivera-Silva

**Affiliations:** 1 Geo-Ocean, Univ Bretagne Sud, Univ Brest, CNRS, Ifremer, UMR6538, F- 56000 Vannes, France; 2 Departamento de Ciencias de la Tierra, Facultad de Ciencias Experimentales, Universidad de Huelva, Campus de El Carmen, Huelva, Spain; 3 Centro Científico Tecnológico de Huelva, Universidad de Huelva, Huelva, Spain; 4 DFG Center for Advanced Studies ‘‘Words, Bones, Genes, Tools: Tracking Linguistic, Cultural and Biological Trajectories of the Human Past’’, Eberhard Karls University of Tübingen, Rümelinstrasse 23, 72070 Tübingen, Germany; 5 UMR 7194 Histoire Naturelle de l’Homme Préhistorique, CNRS, Muséum National d’Histoire Naturelle, Université Perpignan Via Domitia, Paris, France; 6 LR3G, FS, Abdelmalek Essaadi University, 93000 Tétouan, Morocco; 7 Departamento de Ciencias de la Tierra y Física de la Materia Condensada, Facultad de Ciencias, Universidad de Cantabria, 39005 Santander, Cantabria Spain; 8 CNRS, IRD, INRAE, Coll France, CEREGE, Aix Marseille University, 13545 Aix-en-Provence, France; 9 Lixus Archaeological Site, Ministry of Youth, Culture and Communication, Larache, Morocco; 10 Departamento de Geología, Facultad de Geología, Universidad de Oviedo, Campus de Llamaquique, Oviedo, Spain; 11 Centro de Investigación, Tecnología e Innovación (CITIUS), Universidad de Sevilla, Sevilla, Spain

**Keywords:** Anthropology, Palaeontology

## Abstract

Footprints represent a relevant vestige providing direct information on the biology, locomotion, and behaviour of the individuals who left them. However, the spatiotemporal distribution of hominin footprints is heterogeneous, particularly in North Africa, where no footprint sites were known before the Holocene. This region is important in the evolution of hominins. It notably includes the earliest currently known *Homo sapiens* (Jebel Irhoud) and the oldest and richest African Middle Stone Age hominin sites. In this fragmented ichnological record, we report the discovery of 85 human footprints on a Late Pleistocene now indurated beach surface of about 2800 m^2^ at Larache (Northwest coast of Morocco). The wide range of sizes of the footprints suggests that several individuals from different age groups made the tracks while moving landward and seaward across a semi-dissipative bar-trough sandy beach foreshore. A geological investigation and an optically stimulated luminescence dating of a rock sample extracted from the tracksite places this hominin footprint surface at 90.3 ± 7.6 ka (MIS 5, Late Pleistocene). The Larache footprints are, therefore, the oldest attributed to *Homo sapiens* in Northern Africa and the Southern Mediterranean.

## Introduction

Since the discovery of the 3.66 Ma hominin trackways at Laetoli in Tanzania in 1976^1–3^ paleoanthropologists have emphasized the importance of footprint studies. Footprints represent snapshots of life, and provide direct information on the biological (stature, age, body mass), biomechanical (speed, gait) and behavioural features of hominin groups^3–9^.

Over the past 2 decades, a large number of hominin footprint sites have been discovered on different continents^7–19^. Despite an increase in the number of findings, their spatiotemporal distribution is still highly heterogeneous. For instance, in Africa they are mainly concentrated in the east^1–3,7,12,20^ and south of the continent^15,21–23^. In contrast, evidence from North Africa found in Holocene deposits is sparse^4,24^. This fact is relevant considering that this region is central to an understanding of the evolution of hominins. Occupied since 2.4 Ma^25^, North Africa also includes the Jebel Irhoud site where the earliest *Homo sapiens* remains were discovered^26,27^. For more recent periods, the rich Mousterian and Aterian industries^28,29^ testify to various human settlements during the Upper Pleistocene, particularly in coastal environments. In 2022, in the course of a field mission as part of a research project investigating the origin and dynamics of the coastal boulders that litter this rocky coastline in NW Morocco^30^, we identified new hominin footprints on a rocky beach in Larache (NW of Morocco). The site covers an area of about 2800 m^2^ over which have been preserved a large number of tracks and trackways that were documented by both aerial and terrestrial photogrammetry. This discovery presents an opportunity to enhance our knowledge on the patchy ichnological context of the North African coast through recourse to a multiproxy research approach. A prior analysis of the geology, geomorphology, and stratigraphy of the site sets the context for a description of these footprints and inferences on their biological characteristics from morphometric data. The geochronology of the site and the importance of these footprints from a geographical and temporal perspective are then discussed.

## Results

### The rocky beach of Larache (NW of Morocco)

**Figure 1:**
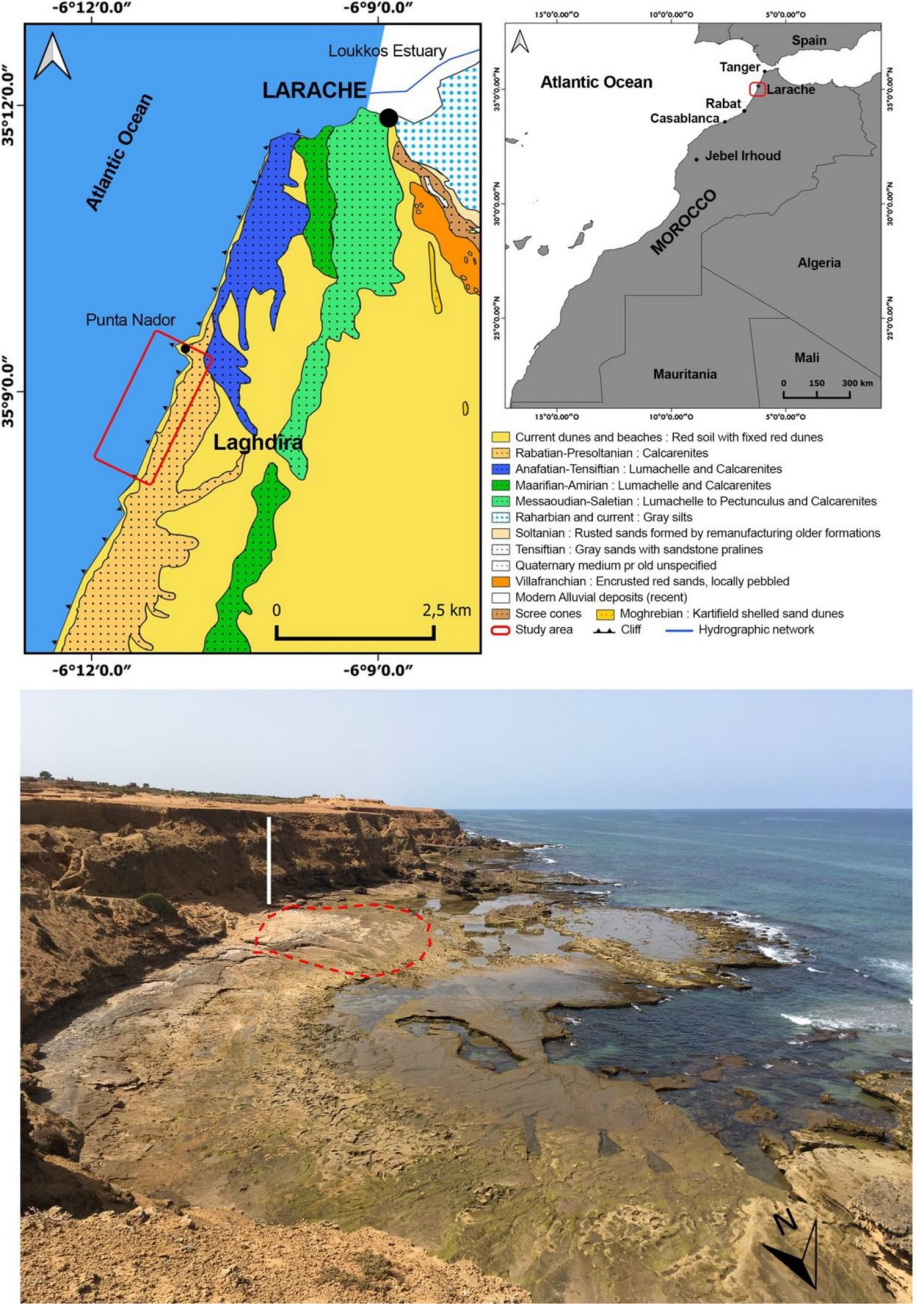
Geological sketch map of the Larache area and panoramic photograph of the rocky shore platform showing the location of the indurated beach. The area delimited by the dotted red line corresponds to the footprint discovery zone. The vertical white line corresponds to the location of the sediment log analyzed in this study. The geological sketch map was generated by QGIS software (v.3.28.4) software (https://www.qgis.org/fr/site/forusers/download.html) based on the Geological Map of Morocco (Scale: 1/1,000,000 N.M.S.G—260—1985).

**Figure 2:**
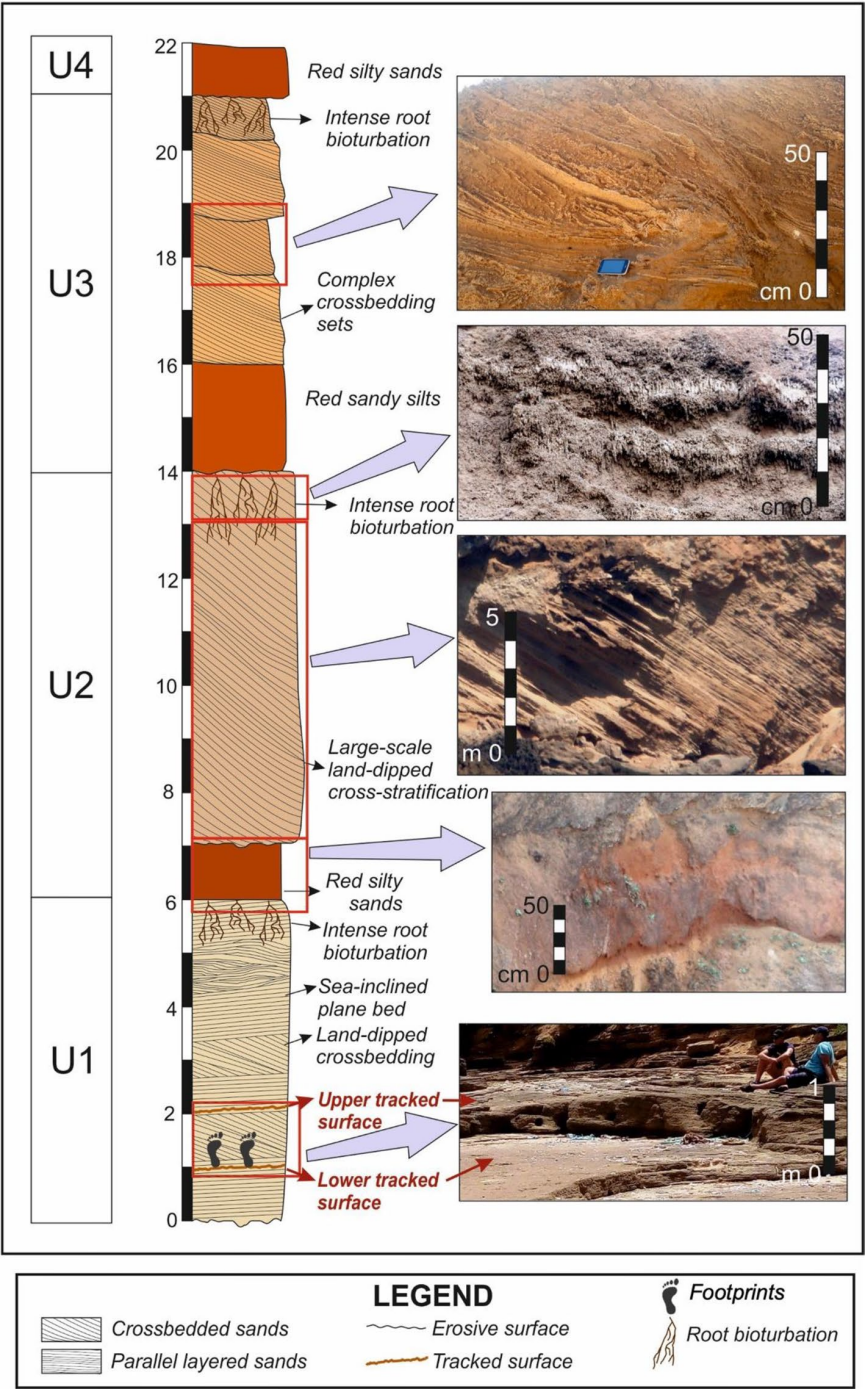
Sedimentary log of Larache site cliff. The right border of the sediment bodies in the log indicates the relative changes in average grain size. The colours of the bodies represent the approximate natural colour of the sediments. Pictures illustrate the aspects of some of the sediment bodies.

Larache is located on the northwestern Atlantic coast of Morocco, south of the Loukkos River estuary and the city of Larache (Fig. 1). The sandy beach to the north of the estuary is made up of fine and medium quartz and bioclastic sand, sometimes covered by dunes up to 10 m high or limited inland by a Quaternary sandstone cliff that constrains the mouth of the Loukkos. The rocky shore platform beach of Larache is situated south of this estuary. The platform is part of the Rmel Plateau which is bounded seaward by actively retreating cliffs with an elevation ranging from 20 to 30 m above sea level. Older inactive cliffs are present 1–3 kms inland. The active cliffs form an almost straight shoreline with a few promontories, including Punta Nador and Laghdira (the northern limit of the study area). At the Larache site, the intertidal width of the platform varies from 20 m at the promontories to 100 m in the widest sectors, with an average width of 60 m. The surface of the platform dips gently seaward or landwards^30^ (Fig. 1). The cliffs consist of a succession of poorly consolidated Quaternary deposits with large foreset beds of aeolianites, thin red sandy edaphogenic levels with continental gastropods, and metric cross-bedded sets of beach deposits^30^ (Fig. 2).

**Figure 3:**
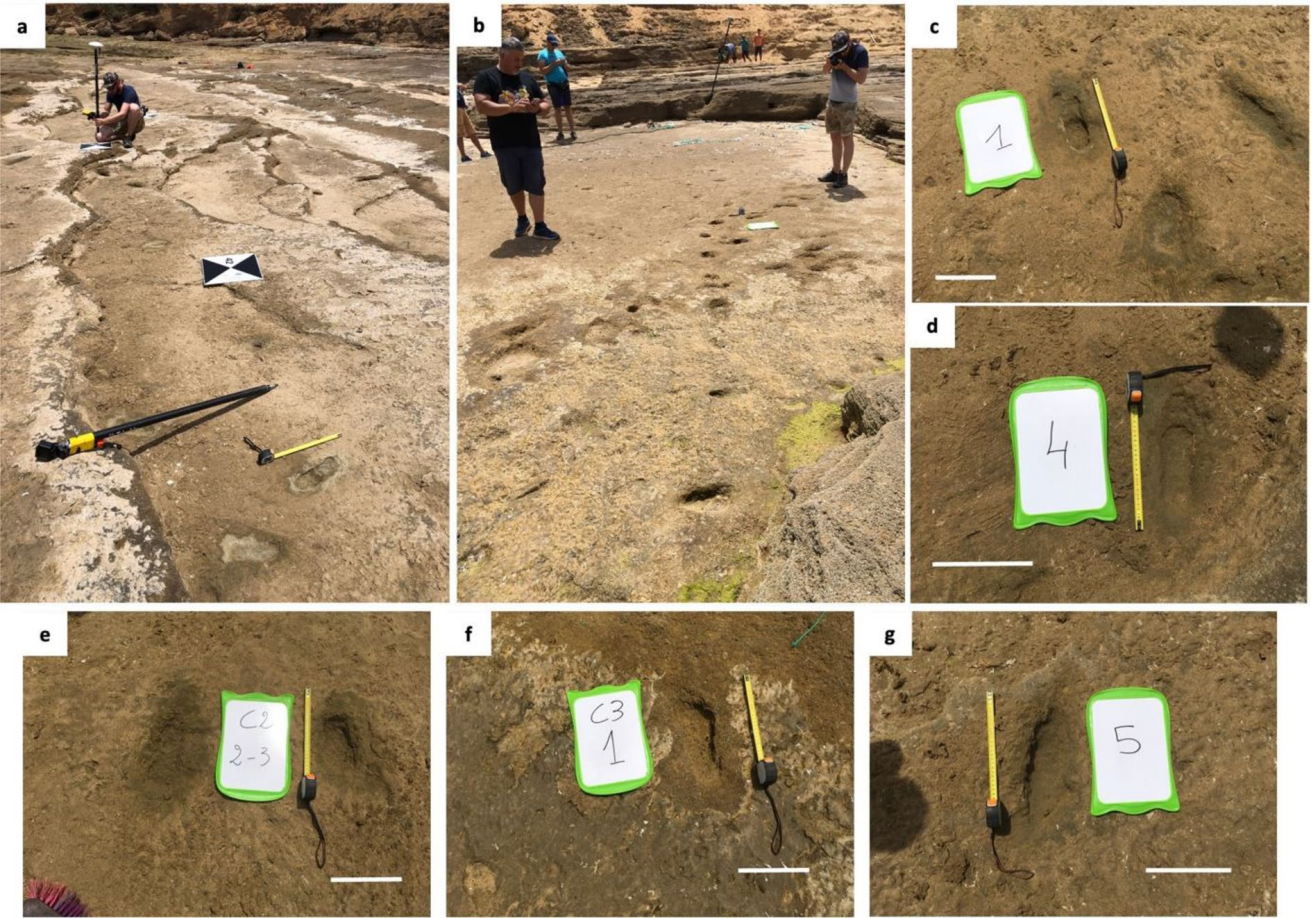
Images of some of the hominin tracks in Larache. (**a**) Two tracks side by side at the bottom of the photo, which also depicts a ground control point (chequered cardboard) for differential GPS surveying. (**b**) Two cross trackways and photography for 3D footprint modelling. (**c**) to (**g**) Detailed view of some footprints. White Scale bars = 20 cm.

**Figure 4:**
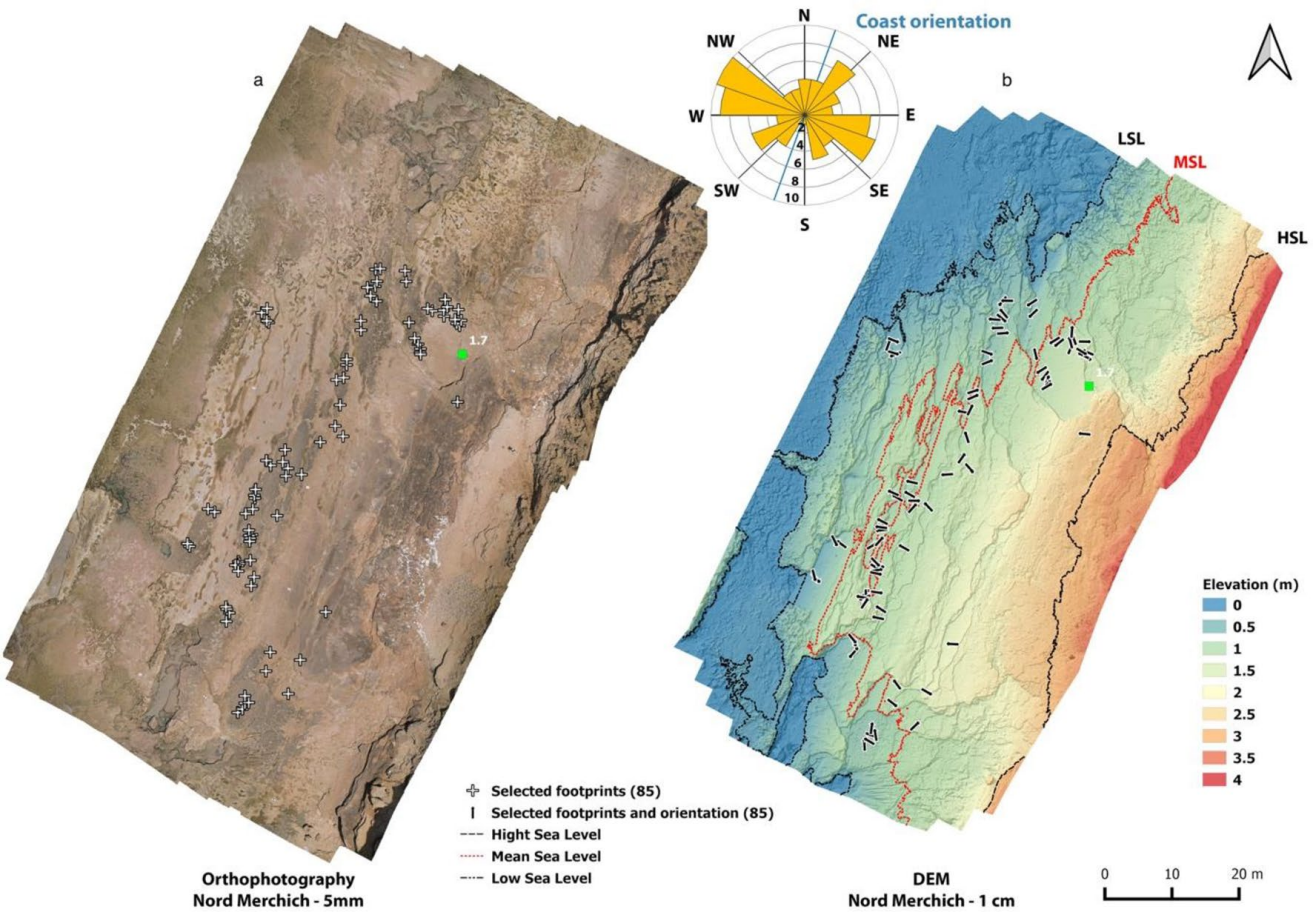
The Larache footprint site. (**a**) Position of the footprints (white crosses) plotted on an aerial photo from a drone flight (orthophotography). (**b**) A Digital Elevation Model (DEM) of the site with the orientation of the footprints. The green square represents the point at which the sediment sample was taken for OSL dating. The coordinates of the sampling point are: (X: 428,456,313—Y: 506,198,258–Z: 1,7). The wind rose shows the preferential direction of the footprints (orientation of the tracks) relative to the coastline. The maps in (**a**) and (**b**) were created using *QGIS* software (v.3.28.4)(https://www.qgis.org/fr/site/forusers/download.html). (**a**) was based on the photogrammetric orthomosaic produced using *Agisoft Metashape* software (version.2.0.1, https://www.agisoft.com/forum/index.php?topic=14904.0). (**b**) is a Digital Elevation Model (DSM) with a resolution of 1.09 cm per pixel which was computed using *Agisoft Metashape software* (version.2.0.1) and imported to a GIS environment using *QGIS software* (v.3.28.4). (**a**) was obtained by a low-altitude programmed flight using an Unmanned Aerial Vehicle (UAV) on July the 5th, 2022. A multirotor Mavic Pro Platinum quadcopter (DJI, Nanshan District, Shenzhen, China) equipped with a 12.35 MP camera was collected at nadirs at an elevation of 12 m.

As these quartz-arenite cliffs retreat, the sandstones on the shore platform surface are exposed (Fig. 1). Overall, the stratigraphy of the Larache site is characterized by the alternation of different types of sedimentary deposits and the ongoing erosion of the cliffs, which has led to the exposure of the underlying sandstones on the shore platform surface^30^. The rocky shore platform sector where we discovered the footprints is geologically interpreted as a fossil beach foreshore and appears to be better preserved from erosion and weathering than the adjoining area, since it is partially protected from the lateral impact of swell on the south coast by a large boulder and frontally by the higher elevation of the platform (Fig. 1). On the north coast, the site is highly eroded, with partial collapse of the platform, likely leading to future weakening and demise of the footprint site (Fig. 1). The footprints (Figs. 3 and 4) are stratigraphically located in the lower part of the Pleistocene succession of the Larache cliffs, being part of its basal unit (Unit 1). The tracked surfaces appear at the top of two sheets of the lowermost visible stratum. The tracked layers are seaward-inclined plane beds, exhibiting isolated or aligned footprints. The sediment sample used for OSL dating was taken from the lower tracked surface to the northeast of the tracks site (Figs. 2 and 3, Fig. S1).

The local geology characterized by a steep coastal cliff is divided into 4 units using sedimentological criteria (Fig. 2). Unit 1 consists of alternating sets of fine sandstones displaying landward-inclined cross-bedded and seaward-inclined parallel laminations. The sandstones are composed of quartz grains of medium-fine sand in a 90% (Fig. S2), but also include some bioclastic grains of bivalve shell fragments, echinoderms and particular benthic foraminifera (*Quinqueloculina* sp.) (Fig. S2). The rock is consolidated by two different types of cements: a primary calcareous cement and a secondary one of siliceous nature. The discovered footprints are part of tracked surfaces inclined seaward but separated by a set of landward-dipping crossbeds. This cross-bedded set wedges towards the sea so that it disappears horizontally by about 6 m (lower picture in Fig. 2). Thus, the tracked surfaces appear superimposed in the westernmost area and would be considered as two sheets of the same stratum. The lowermost part of this unit comprises of a 2 m-thick layer of more compacted horizontal laminated shelly sandstones, with low porosity. The levels of Unit 1 under these shelly sandstones are not visible. This unit includes the surfaces tracked by hominins. The OSL-dated tracked layer yielded an age of 90.3 ± 7.6 ka (see Table 1 and “Methods” section). Unit 2 starts with a metric edaphic level of red silty sands. However, most of the unit consists of a 7 m-thick set of complex cross-bedded deposits that culminates in a strongly root-bioturbated level. This complete unit can be interpreted as an aeolianite. Unit 3 is similar to unit 2. It starts with a meter-thick pedogenic level of red sandy silts and continues with 6 m of several sets of curved-base cross beds. Many of the cross-bedding sets are deformed, possibly by seismic activity. The last of these levels is also strongly bioturbated by roots. This unit has also been interpreted as an aeolianite. Unit 4 consists of a metric stratum of red silty sands of pedogenic origin and is overlain by Holocene dunes.

**Table 1:** Summary of the results and parameters used for OSL dating calculations.

**Sample**	Larache 5568
**No. of aliquots considered for burial dose**	42
**Altitude (m)**	1.7
**Overburdern density (g/cm**^**3**^)	1.8
**Depth (m)**	0.01
**Water content (%)**	13 ± 3
^**40**^**K (Bq/kg)**	40.3 ± 4.0
^**232**^**Th (Bq/kg)**	2.8 ± 1.1
^**238**^**U (Bq/kg)**	4.9 ± 4.6
^**40**^**K (%)**	1.114 ± 0.012
^**232**^**Th (ppm)**	0.70 ± 0.26
^**238**^**U (ppm)**	0.38 ± 0.35
**Water corrected beta dose rate (Gy/ka)**	0.122 ± 0.019
**Water corrected gamma dose rate (Gy/ka)**	0.091 ± 0.019
**Cosmic dose rate (Gy/ka)**	0.250 ± 0.025
**Overdispersion (%)**	19.6
**Dose rate (Gy/ka)**	0.463 ± 0.0.40
**Burial rate (Gy)**	41.8 ± 1.3
Age (ka)	90.3±7.6

Lab code from the Servicio de Radioisótopos (CITIUS, University of Seville).

### Ichnological assemblage

The laterality of 91% of the 85 footprints identified at Larache (Fig. 4), yields 30 (35%) as left and 39 (46%) as right footprints (Table S1). This assemblage contains isolated footprints as well as trackways, including 2–6 footprints.

The footprints are preferentially oriented in a WNW-ESE direction (Table S1), relatively perpendicular to the present shoreline, and secondarily in a SW–NE direction (Fig. 4).

**Figure 5:**
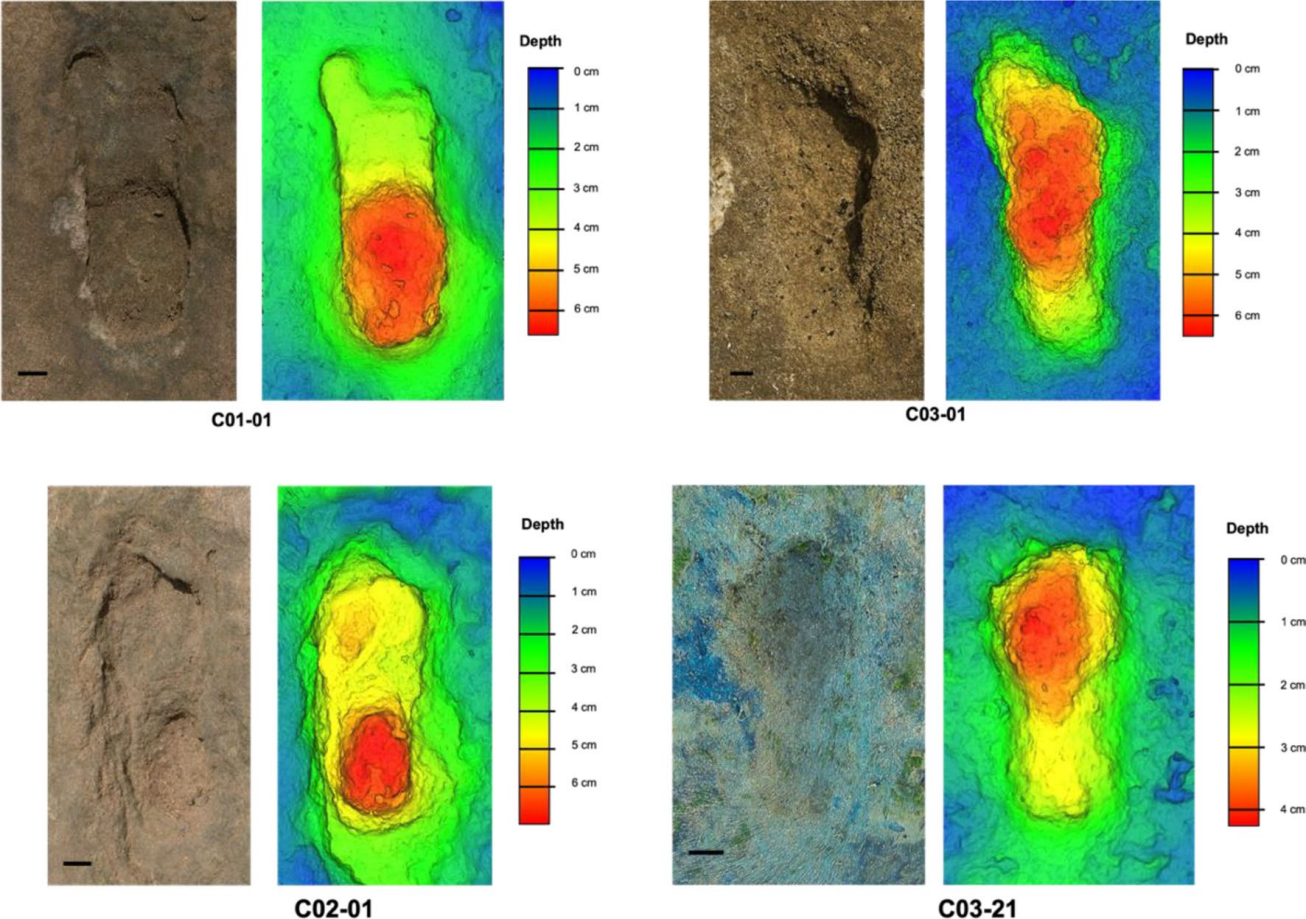
Hominin footprints from Larache. (**a**) Natural views and shaded 3D elevation of (**a**) C01-01, (**b**) C03-01, (**c**) C02-01 and (**d**) C03-21). Scale bar, 2 cm. 3D models were generated with *Agisoft Metashape* software (version.2.0.1, https://www.agisoft.com/forum/index.php?topic=14904.0) using between 18 and 20 images taken with a Nikon D7500 (20.9 MP, Nikon AF-S DX35mm f/1.8 G) from a height of approximately 0.5–1.5 m.

The morphology of the Larache footprints is variable, a phenomenon regularly observed particularly in coastal or dune contexts^7,9,13,16,18^. Indeed, footprint morphology is not only impacted by the biological and biomechanical features of the track-makers but also by the nature of the substrate and the taphonomic agents^4,31,32^. Despite this extramorphological variation, the Larache footprints reflect human foot characteristics such as a rounded heel and an adducted hallux. The toe impressions can be difficult to discern from the rest of the footprint, which has also been observed at other sites with similar depositional conditions^9,13,18^. However, the hallux is distinguishable on some footprints (Fig. 5—e.g., C1-01). The depth distribution is also variable, although trends can be observed: the area associated with the heel and the forefoot are the deepest. The deepest area of the heel is generally located in its middle. The deepest area of the forefoot is usually located in the areas associated with the most medial metatarsal heads or toes (hallux and second toe). This depth distribution is consistent with the distribution of plantar pressures during human walking. During the stance phase, the heel is the first part to contact the ground (“heel strike”). The weight is then transferred to the forefoot, and in particular, the metatarsal heads and then the toes (especially the most medial) during the propulsion^33,34^. Finally, a difference in depth can be observed depending on the footprint dimensions. The smallest footprints are shallower than the longest ones (Fig. S3).

**Figure 6:**
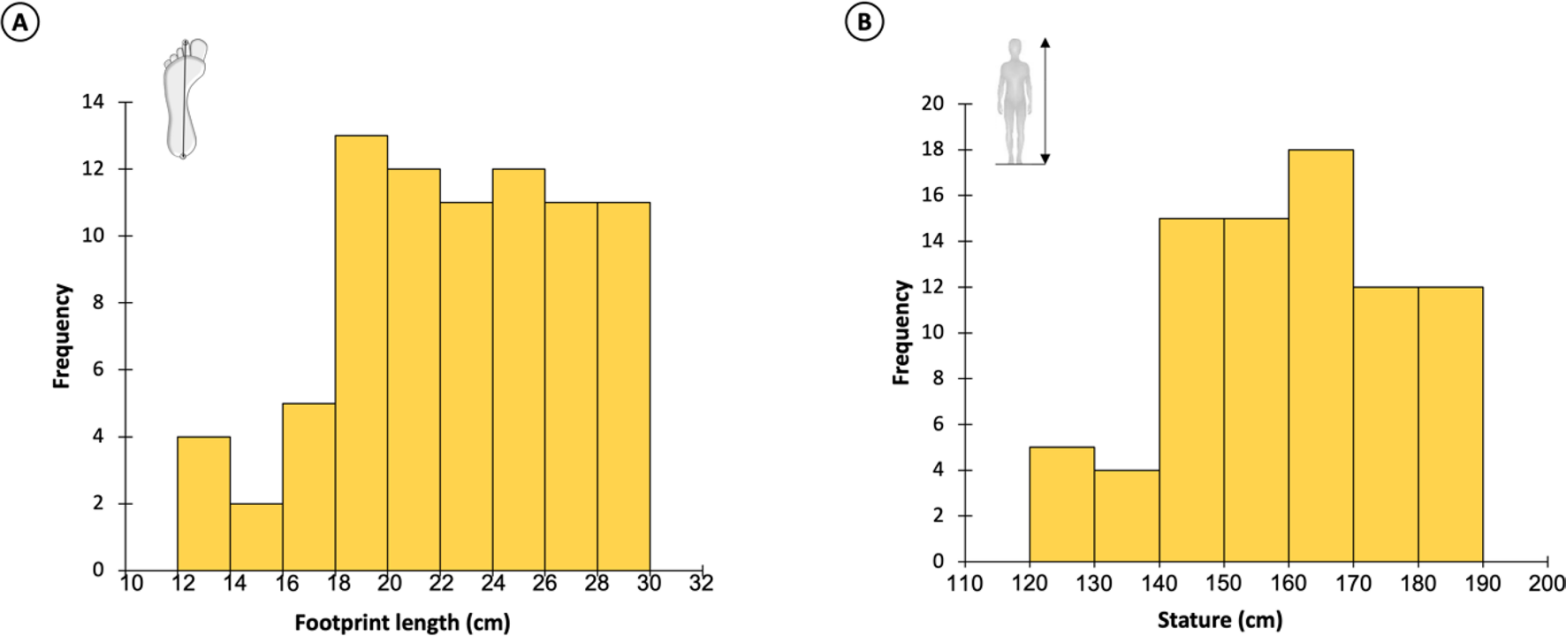
Paleobiological estimates based on 81 longitudinally complete footprints from Larache. (**A**) Distribution of footprint lengths. (**B**) Distribution of estimated statures.

The length was measured for 81 of the 85 footprints (Table S1). It ranges from 12.7 to 30.0 cm (mean: 22.7 cm) following an asymmetric normal distribution (Shapiro–Wilk: *p* value > 0.05). Footprints longer than 20 cm are more numerous (80%) than those shorter than 20 cm (20%). These lengths correspond to estimated statures between 120.8 and 189.0 cm (average: 160.0 cm). Most of these footprints (89%) have an estimated stature higher than 140 cm (Fig. 6).

**Figure 7:**
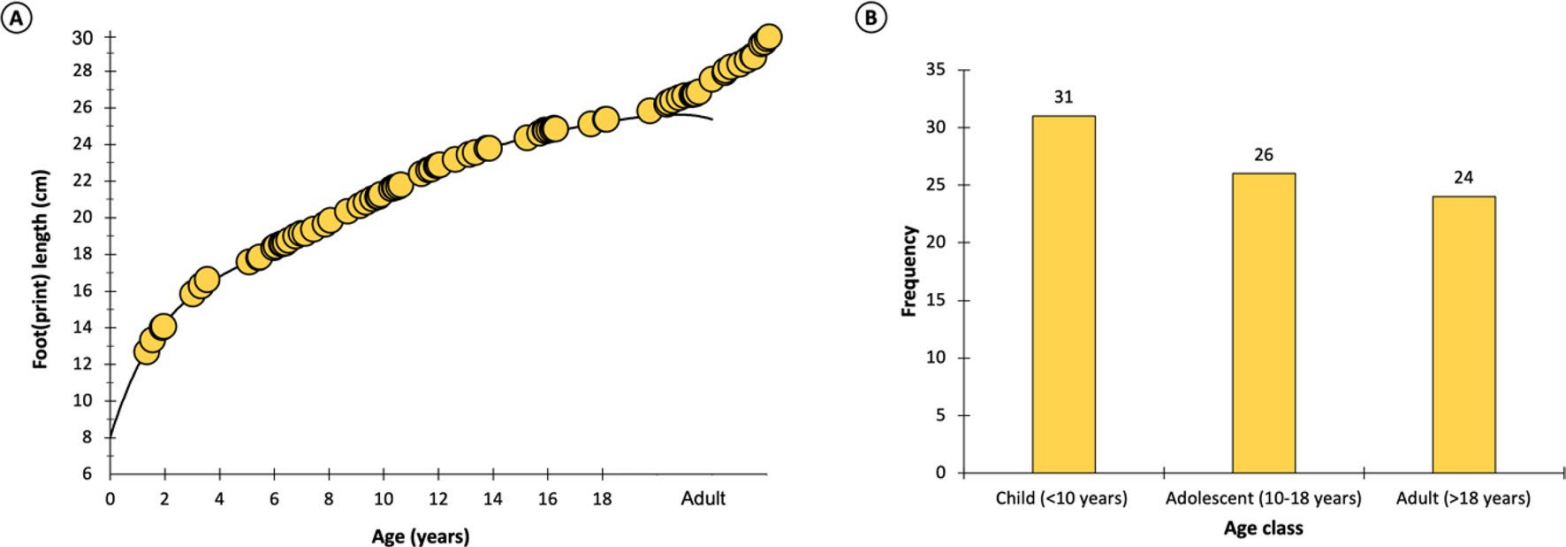
Paleobiological estimates based on 81 longitudinally complete footprints from Larache. (**A**) Position of the footprint lengths on a curve representing the stature variation with age. (**B**). Distribution of estimated age classes.

The lengths correspond to the three age classes: children, adolescents and adults. The distribution of the footprints according to these three age classes is also relatively balanced (Fig. 7). There are slightly more footprints attributed to children (31) than to adolescents (26) or adults (24). However, the model used to estimate age classes has some uncertainties based on the average foot length for each age (cf. “Methods” section). Thus, footprints associated with older children may have been made by adolescents, and vice versa. The same uncertainty exists for the footprints close to the boundary between adolescents and adults (cf. Discussion).

These palaeobiological estimates of stature and age are made for each footprint and not for each individual. Although part of the assemblage is composed of isolated footprints, it is possible to estimate a minimum number of individuals from the experimental knowledge of intraindividual variation (cf. “Methods” section). The 81 measured footprints correspond to at least 5 individuals. Among this minimum of 5 individuals, there is a young child (between 1 and 4 years), an older child (between 4 and 8 years), an adolescent or small adult (148.8–161.8 cm), a medium-sized adult (162.9–175.9 cm) and a tall adult (176.2–189.0 cm) who is likely male based on the sexual dimorphism of height known in fossil and extant *Homo sapiens*^35,36^.

## Discussion

The sedimentological features of the tracked layers are evidence informing about the environment in which footprints were made. Unit 1 is composed of a rhythmic alternation of two different primary sandy facies. Facies of land-dipped cross-beds and facies of sea-inclined parallel lamination. The sediment that constitutes both facies is a medium-to-fine sand, mainly of quartz, but also including bioclastic grains. The grains are partially compacted by calcareous and silica cements. The grain size, gentle slope, and conjunction of these facies in the form of a sequence is the same as that identified on the foreshore zone of a semi-dissipative beach with transverse landward migrating bars and troughs^37,38^. Thus, the landward-inclined crossbedding represents the migration of the bars. In contrast, once a bar becomes attached to the beachface, a seaward-inclined slope is generated, wherein the waves build up parallel sheets of sand inclined in the same sense until the arrival of a new bar.

In the Larache case, the footprints were probably left: (1) in the course of fair-weather wave conditions (no storm surge), at the limits of swash flow (2) and the landward limits of the spring high tidal zone over the beach foreshore bar (3), three conditions that would have corresponded to limited swash flow duration and very thin laminar swash flow, all potentially the most favorable conditions in the course of the spring-neap tidal cycle to salt-crusting and inception of preservation. These conditions correspond to a situation where the footprints left on the upper beach were not washed out by vigorous swash on the beach face and have time for the salt-crusting that we deem as initiating the preservation process. It is interesting to note that the tracked surfaces are seaward-inclined whereas these surfaces are covered and preserved by sets of landward cross-beds. The presence of bioclastic shell fragments indicates a marine contribution to the essentially terrigenous beach quartz sand. The presence of a primary calcareous cement suggests the possibility of the occurrence of an early cementation that ensued in the same sedimentary environment and forming a hardened beach. This process could contribute to the final preservation of the footprints. The dependence of footprints preservation essentially on taphonomic processes that potentially preserve bedforms, such as the afore-mentioned early cementation (of the sediment or within the microbial mat), rapid covering by sediment, and overgrowth by microbial mats has been well-described^39^. According to Thulborn^40^, footprints are most commonly preserved in environments that experience periodic or cyclic sediment accumulation as observed in our case. In the Larache case, the mobility of the bars covered up footprint-bearing surfaces located within water-saturated troughs separating the bars. This is observed in other coastal environments with similar dynamics^41–45^.

Considering the OSL dating, the estimated age for this track site is 90.3 ± 7.6 thousand years (ka) (MIS 5c, formerly known as the "Ouljian" throughout Morocco). Studies on sea-level fluctuation, geomorphological and sedimentary context of the Larache coastal zone are sparse. Pierre et al.^46^ referenced an Ouljian outcrop situated approximately 1 m above the present sea level on a cliff north of the Loukkous estuary. In contrast, in a description the currently active cliffs south of Larache, Adil^47^ mentioned the presence of the Ouljian at the base of these cliff but did not specify a date or chronological context. To the north of Larache (Tangier Region), El Kadiri et al.^48^, and El Abdellaoui et al.^49^, have predominantly focused on dating MIS 5 marine terraces (between MIS 5a and MIS 5e), while underscoring the influence of neotectonics in uplifting sedimentary facies of the Gibraltar arc by approximately 0.06 mm/year to 0.1 mm over the last 100,000 years. This corresponds to an elevation of 7–10 m. South of Larache, between Rabat and Casablanca, MIS 5 sea-level oscillations have been extensively documented. Weisrock^50^ and Chahid et al.^51^ placed sea level during MIS 5c at around − 20 m above the present level. This MIS 5c level is observed at various elevations between present sea level and + 6 m. It was marked in its final phase by the formation of a high continuous aeolian sandy ridge dated between 103.8 ± 8.2 and 93.3 ± 7.1 ka^51^. The chronostratigraphic evidence both north and south of Larache is consistent with the dating of the sedimentary level on which the tracks were established on a beach foreshore environment, subsequently preserved in the medium and long terms by the formation of a dune. However, the impact of neotectonics and the rate of sedimentary facies uplift at our site remain to be determined. Using this dating for estimating the coastal position when the tracks were recorded is feasible. Precise dating of the indurated platform would throw more light on the paleoenvironmental context and deposition of coastal and marine sediments in this region.

The discovery of the Larache footprints represents further evidence of the importance of North Africa, and the Moroccan region in particular, during hominin evolution. Occupied by hominins since at least 2.4 Ma^25^, North Africa has notably yielded the earliest occurrences of *Homo sapiens*: the 315,000-year-old fossils of Jebel Irhoud^26,27^. Occupations in Morocco have remained important during the most recent periods, including the Middle Stone Age, during which the Larache footprints were left. Fossil remains, and notably, the rich Mousterian/Aterian industries testify to the presence of hominins in the Atlantic and Mediterranean coasts during this period^52,53^. The contribution of recent studies has considerably clarified our knowledge of the chronology of these records. The western Atlantic coast between Rabat and Tangier, where the Larache footprints are located, was occupied in several places. A large part of these occupations is in the Rabat-Temara region, where paleoanthropological remains and archaeological artefacts attest to occupations by *Homo sapiens* between 150 and 50 ka ago and notably during MIS 5^54–56^, when the Larache footprints were made.

From an ichnological point of view, the Larache footprints represent an important discovery. Indeed, no other site in North Africa has yielded footprints dating from the Pleistocene or Pliocene. They are, therefore, the oldest human footprints in this region and among the oldest footprints attributed to *Homo sapiens* worldwide. Only two other regions have yielded older *Homo sapiens* footprints: South Africa, with several coastal sites, the oldest of which is dated to 150 ka ago^15,23^, and the Arabian Peninsula, where footprints dated to 120–110 ka ago were discovered at Alathar^16^. The site of Larache is also important for the number of footprints that have been discovered. Indeed, the ichnological assemblage, including 85 footprints, is important. Most sites, including those mentioned above, have yielded, in the best of cases, less than a few dozen footprints^57^. The absence of archaeological material (animal bones, tools, occupation structures) or paleoanthropological material (hominin skeletal remains) is another feature of the Larache ichnological assemblage. However, such an association is extremely rare within the fossil hominin footprint record^57^. Footprints from Melka Kunture^7^, Schöningen^14^ and Le Rozel^9^ are among the only ones to be associated with archaeological material. In the vast majority of cases, ichnological assemblages are not associated with any other type of material.

One of the most original aspects of footprints is that they provide access to very brief periods of life, like snapshots^20,58^. They can provide information not only on the composition of groups but also on their behaviour^6,8,9,18,59^. In Larache, the morphometric study shows that a group of young children, adolescents and adults left these prints with a minimum number of 5 individuals. The 81 prints analysed represent a balanced distribution between children, adolescents and adults. Although there is no doubt that the track-making group was composed of different age classes (children, adolescents and adults), it is necessary to exercise caution when making inferences about the group's composition from estimated statures and age classes. First of all, these estimates are based on the morphology of the footprints, which varies even if a single individual makes them. The intra-individual morphological variation can be significant, particularly in dune environments where footprints are left in soft substrates^32,60,61^. It is, therefore, necessary to be cautious when estimating biological characteristics from footprints, especially when they are isolated and intra-individual variation cannot be quantified. Indeed, in the case of isolated footprints, the difference between estimated and actual stature can exceed 10%^61^. In addition to this uncertainty linked to intra-individual morphological variation, there is also uncertainty linked to taphonomic modifications. Although footprints discovered in the fossil record are rapidly covered, they can be eroded by taphonomic agents, thus modifying their morphology and consequently potentially biasing palaeobiological inferences^62^. Secondly, age class estimates are made from an average variation between foot length and age obtained for different modern populations. This average variation is not perfect since the attribution of age classes for some individuals may be erroneous, particularly for individuals whose footprint lengths are close to the limits between two age classes. In the case of the Larache footprints, many are close to the limit between children and adolescents. Thus, some footprints attributed to adolescents could have been left by children and vice versa. An identical uncertainty is present for the limit between adolescents and adults. Furthermore, the relative distribution obtained may also overestimate the number of children for biometric reasons. Indeed, children need to make more strides to cover the same distance as an adult, leaving more footprints. Lastly, the composition reflected by the footprints is not necessarily that of the entire social group that occupied the area. Only part of the social group may be present, as the performance of certain tasks may have led to the selection of individuals^5,6,59^.

A key question when studying footprints is what the individuals were doing at the site. Since no occupation structures were found, this site may correspond to a passage and/or foraging site. While Pleistocene *Homo sapiens* were hunter-gatherers, individuals likely left the Larache footprints while probably searching for resources. Numerous archaeological discoveries, particularly in Morocco and notably in the Rabat-Temara region, have shown the importance of coastal areas for access to resources, whether raw materials, prey or even plants^52,63–66^. In this context, the preferential orientation of the Larache footprints towards the offshore could maybe indicate the search for marine resources. The presence of young children, in Larache, possibly contributing to the search for these resources, could provide unique information on the social behaviour of Pleistocene *Homo sapiens* populations. However, further studies will be needed to validate this hypothesis.

## Conclusion

The Larache coastal hominin tracksite reported here is dated to 90.3 ± 7.6 ka and represents one of the world’s largest and best-preserved Late Pleistocene tracks sites, and the sole documented site in North Africa and Southern Mediterranean. Morphological characteristics undoubtedly relate these tracks to hominins, and due to their geological age and geographical location, they belong to *Homo sapiens*. According to the morphometric comparisons made with the complete footprints, the statures of the track-makers vary from 120.8 and 189.0 cm (mean: 160 cm), and the tracks to a minimum of 5 individuals. The distribution of the footprints with respect to the shoreline provides a snapshot of the movements of multigenerational *Homo sapiens* individuals (children, adolescents, and adults) in this site. The distribution supports the ecological relationship between *Homo sapiens* populations and coastal areas where no other type of anthropological or archaeological evidence has yet been found. The protection of the Larache site from intense marine erosion until now has enabled the intact preservation of the traces. However, the ongoing collapse of the rocky shore platform in the northern zone of the site could lead to its eventual demise, and that of the tracks it has preserved thus far. The loss of the site in the medium and long term is likely to be caused by rising sea level and storm events. In the short term, other new footprints will be discovered as sediments are eroded. It would be interesting to monitor this erosion to expose new, complementary tracks that could be used to clarify hominin group size. The various caves along Larache's southern coastline should also be explored for any fossils or lithic traces that might be present.

## Methods

### Geological context and sedimentary analysis

The geological context was determined by standard field mapping of exposures and outcrops. The detailed sedimentary column of the sequence in which the track’s surface is preserved has been recorded, including dip direction and facies description through analysis of the grain characteristics (Fig. 2).

### Footprint analysis

#### Footprint identification

The identification of the footprints was based on the anatomical characteristics of the human foot: a rounded heel, a plantar arch, relatively short toes and an adducted hallux^4,67^.

#### Footprint recording

Descriptions, measurements, and photos of footprints were made directly in the field (Fig. 2). We carried out a low-altitude UAV flight of the footprint surface on the indurated shore platform and a series of DGPS measurements of the most visible tracks and the limits of footprint surfaces (clusters). UAV images were collected at nadirs along calibrated flight paths parallel to the coast, at an elevation of 12 m using a Mavic Pro Platinum quadcopter (DJI, Nanshan District, Shenzhen, China) equipped with a 12.35 MP camera. DJI GS Pro Flight Planner software™ was used for programming the automatically and autonomously executed flight plan. High-overlap image sets ensure sufficient target points in adjacent images as the standard procedure; we planned the flight paths to provide 80% overlap between successive images and 70% sidelap between adjacent paths. A total of 461 photographs were collected over the study area. We used Agisoft Metashape Professional software (v.1.6.2) to process the images. During the flight survey, 12 ground control points (GCPs) consisting of black-and-white chequered cardboard, essential to produce georeferenced aerial images, were deployed at different locations on the shore platform to provide the widest possible coverage (Fig. 3a). Their positions were recorded using a Trimble differential GPS in real-time kinematics mode (Fig. 3a). Digital elevation models (DSM) with a resolution of 1.09 cm per pixel and orthophotographs with a resolution of 5.43 mm per pixel were computed using Agisoft Metashape software and imported to a GIS environment using QGIS software (v.3.28.4) to characterize the spatial distribution and orientation of the footprints. The data collected during this flight were used to characterize the spatial distribution and orientation of the footprints using QGIS software (v.3.28.4). In addition, 28 footprints were scanned by close-up photography and vertical mosaic of high-resolution digital images taken with a Nikon D7500 (20.9 MP, Nikon AF-S DX35mm f/1.8 G) from a height of approximately 0.5–1.5 m (Fig. 3b). Photographs were photogrammetrically modelled using *Agisoft Metashape* software (v.2.0.1) to obtain 3D models of the best-preserved footprints.

#### Morphometry

The lengths were measured in 3D along the longitudinal axis of the footprints. For a complete footprint, this length corresponds to the distance between the base of the heel and the tip of the second toe. The measurements taken from the 3D models were compared to those obtained directly in the field and those measured on the orthomosaics obtained with the drone (Table S1). No significant difference was observed between the measurement techniques (Kruskal–Wallis: *p* value > 0.05; mean difference < 0.1 cm).

#### Stature estimates

Stature (S) is a variable commonly estimated from footprint length (L). This estimate is based on the strong relationship between foot length and stature (between 13 and 16%). One stature was estimated for each Larache footprint whose length was measured using two published experimental regressions. Both experimental studies were carried out under substrate conditions close to those found at Larache. The first regression equation (S = 3.8 × L + 73.3, r = 0.78)^68^.The second regression Eq. (4.1 × L + 67.6, r = 0.69)^68^. The stature presented in the results section corresponds to the average of the estimates obtained by these two equations. The differences in stature between these two regressions for the Larache footprints sample are minor (mean: 1.1 cm) (Table S1).

#### Age class estimates

An age was also estimated from the length of the Larache footprints. To do this, a curve representing the growth in foot length (Fig. 7, Fig. S4) in modern populations was realised from published data^69–74^ including almost 12,000 individuals from different geographical areas (America, Europe, Africa, Asia). The Larache footprints were positioned on the curve according to their length. An age class was then estimated for each footprint based on its position on the curve.

#### Minimum number of individual estimates (MNI)

Although the footprint assemblage is *partly* composed of isolated footprints, it was possible to determine a Minimum Number of Individuals. For this, we used the intra-individual variation of footprints. Actually, footprints of a single individual in a soft substrate have different sizes and shapes. However, this intra-individual morphological variation is limited; it is possible to use this limit for a sample of isolated footprints to determine a minimum number of individuals. Several studies on experimental or Holocene footprints have shown that this intra-individual variation can be important; the deviation from the individual mean can exceed 10%^4,32,61,68^. Therefore, we used the same assumption as Ashton et al.^13^ in their study of Happisburgh footprints by considering that a single individual could have made footprints falling within ± 10% of each other.

### Luminescence dating

#### Sampling method

A rock sample was extracted from a zone of sedimentary continuity on the surface of the footprints for OSL dating. This sampling was carried out with all possible precautions and following the sampling protocols for OSL dating. A rectangular sample measuring approximately 15 cm × 15 cm was extracted by hand using a hammer and chisel and packed directly into an opaque PVC box. The sample was collected at the lower tracked surface (See Fig. 2, Fig. S1). The coordinates of the sampling point are: (X: 428,456,313—Y: 506,198,258–Z: 1,7). A fragmentation of this sample was used for analysis by transmitted light microscopy (Fig. S2).

#### Sample preparation procedure

The outer layer of the sample was removed to a depth of 25 mm on each side to ensure that the material exposed to sunlight was effectively eliminated. Subsequently, the core of the sample was carefully crushed and wet sieved to obtain grain fractions of 90–180 μm and 180–250 μm. The 180–250 μm fraction was chemically treated to isolate the quartz grains. Initially, it was exposed to a 10% HCl solution until the carbonates were completely dissolved. Following this step, H_2_O_2_ was employed to remove any organic matter present. Two density separation processes were then carried out using sodium polytungstate to separate quartz from feldspar and other heavier minerals effectively. Finally, a 40% HF solution was employed to remove any remaining feldspar residuals and for etching the outer layer of quartz, which might have been influenced by alpha radiation. Lastly, the resulting quartz grains were carefully dried and sieved once more, with the specific fractions falling within the 180–250 μm range selected for subsequent measurements.

#### SAR procedure

Samples were measured using the SAR (Single Aliquot Regenerative dose) procedure^69^. A portion of 180–250 μm quartz grains from the sample was divided into aliquots that were measured individually. Table 2 shows the SAR protocol used. The OSL signal was measured for 40 s at 0.1 s per data point, giving 400 data points (Fig. S5). OSL signal was measured using a blue LED light source. OSL signal was collected with a reading temperature of 125 °C. The signal was dominated by the fast component, considering the first 5 data points (0.5 s) for measurement. The background was calculated considering the end part of the spectrum (50 data points) and subtracted from the measured signal. No feldspar contamination was detected using IR stimulation. Figs. S6 and S7 have been chosen as representative of the OSL measurement process. They show the OSL decay and dose–response curves obtained from the sample.

**Table 2:** SAR Protocol.

Step	Treatment	Measurement
1	Give regenerative dose	–
2	Preheat (220 °C, 10 s)	–
3	OSL (Blue at 125 °C, 40 s)	Lx
4	Give test dose	–
5	Cutheat (180 °C, 10 s)	–
6	OSL (Blue at 125 °C, 40 s)	Tx
7	Cleanout (280 °C, 100 s)	–
8	Return to step 1	–

The resulting populations are normally distributed with overdispersion values < 20% (Fig. S8). Central Age Model (CAM)^70^ has been applied to calculate the equivalent dose (i.e., the accumulated dose due to the ionising radiation received by the quartz grains over the period they have been buried) of each sample.

#### Pre-heat dose recovery test

A pre-heat temperature of 220 °C was considered for the OSL measurements. A pre-heat dose recovery test was conducted for the sample to determine this temperature. Fifteen aliquots were bleached using a daylight simulator for 5 h and subsequently irradiated with 51.6 Gy, previously determined by a dose range test. Five sets of 3 aliquots each were preheated at 5 different temperatures (180 °C, 200 °C, 220 °C, 240 °C and 260 °C). Then, the given/recovered dose ratio was determined for each preheat temperature. The best averaged given/recovered dose ratio was obtained for 220 °C.

#### OSL measurement and analysis

OSL measurements were conducted using the TL/OSL reader Risø TL-DA 20. This instrument is equipped with a calibrated ^90^Sr/^90^Y beta source, which delivers an approximate dose rate of 0.09 Gy/s at the location of the sample disc.

The OSL signal was collected for each measurement from small quartz multi-grain aliquots (30–60 grains) ranging from 1 to 2 mm in diameter. A total of 48 aliquots were measured to ensure accurate and reliable results (Fig. S8).

#### Dose rate calculations

The dose rate for the sample was calculated from the mean activity concentrations of ^40^K, ^232^Th and ^238^U, which were measured by high-resolution gamma spectrometry. Conversion factors were used according to Guérin et al.^71^. The water content considered is indicated in the results (Table 1). It was calculated as the average value between the sample's water content measured at the laboratory and a reference value for the saturation of the sand layers (25%), as the sample was submerged by the effect of the tides for part of the year. The dose attenuation contribution was determined considering this value. The contribution of cosmic radiation to the total dose rate has been calculated as a function of latitude, longitude, altitude, burial depth and mean density of the coating based on data from Prescott and Hutton, 1994^72^. Alpha grain-size attenuation was determined according to Brennan et al.^73^. According to Guérin et al.^71^ beta grain-size attenuation was determined. The minimum and maximum etch depths were 8 and 10 microns, respectively. Beta etch depth attenuation factor was used according to Bell^74^.

#### Dating results

The age of the sample was determined by applying the following expression:

$$Age\;({\text{ka}}) = \frac{{Equivalent\;dose\;({\text{Gy}})}}{{Dose\;rate\;({\text{Gy/ka}})}}$$

where the equivalent dose is expressed in Gray (Gy) and the dose rate in Gray per kilo-year (Gy/ka). The age is therefore expressed in kiloyears (ka) (Table 1). Luminescence dating basics can be found in Aitken^75^.

#### Dating measurement uncertainty

To estimate the measurement uncertainty, the contributions of the standards, the measurement method, the environmental conditions and those derived from the testing process were considered. The values obtained correspond to the time at which the measurements were made. The expanded measurement uncertainty was expressed with a coverage factor k = 2, which, in a normal distribution, corresponds to a coverage probability of approximately 95%. The measurement uncertainty was determined according to JCGM 100:2008^76^.

### Supplementary Information

Supplementary Information.

## Data Availability

All data generated or analysed during this study are included in this published article [and its supplementary information files].
