# Supplementary material for: A Late Pleistocene hominin footprint site on the North African coast of Morocco
Source: Sci Rep. 2024 Jan 23;14:1962. doi: 10.1038/s41598-024-52344-5 (PMC10806055; doi:10.1038/s41598-024-52344-5)
Supplement: Supplementary file 1 — Supplementary Information. [file 41598_2024_52344_MOESM1_ESM.docx]

**Supplementary Information:**

**A Late Pleistocene Hominin footprint site on the North African Coast of Morocco**

**M. Sedrati^1*^. J.A. Morales^2.3^. J. Duveau^4.5^. A. El M’rini^6^. E. Mayoral^2.3^. I. Díaz‐Martínez^7^. E.J. Anthony^8^. G. Bulot^1^. A. Sedrati^9^. R. Le Gall^1^. A. Santos^10^. J. Rivera-Silva^11^**

^1^Geo-Ocean. Univ Bretagne Sud. Univ Brest. CNRS. Ifremer. UMR6538. F-56000 Vannes. France

^2^Departamento de Ciencias de la Tierra. Facultad de Ciencias Experimentales. Campus de El Carmen. Universidad de Huelva. Huelva. Spain

^3^Centro Científico Tecnológico de Huelva. Universidad de Huelva. Huelva. Spain

^4^DFG Center for Advanced Studies ‘‘Words. Bones. Genes. Tools: Tracking Linguistic. Cultural and Biological Trajectories of the Human Past’’. Eberhard Karls University of Tübingen. Rümelinstrasse 23. 72070 Tübingen. Germany

^5^UMR 7194 Histoire Naturelle de l’Homme Préhistorique. CNRS. Muséum National d’Histoire Naturelle. Université Perpignan Via Domitia. Paris. France

^6^LR3G. FS. Abdelmalek Essaadi University. Tétouan 93000. Morocco

^7^ Departamento de Ciencias de la Tierra y Física de la Materia Condensada. Facultad de Ciencias. Universidad de Cantabria. 39005 Santander. Cantabria. Spain

^8^CNRS. IRD. INRA. Coll France. CEREGE. Aix Marseille University. 13545 Aix-en-Provence. France

^9^Lixus archaeological site. Ministry of Youth. Culture and Communication – Larache. Morocco

^10^Departamento de Geología. Facultad de Geología. Campus de Llamaquique. Universidad de Oviedo. Oviedo. Spain

^11^Centro de Investigación. Tecnología e Innovación (CITIUS). Universidad de Sevilla. Sevilla. Spain

*Email: [mouncef.sedrati@univ-ubs.fr](mailto:mouncef.sedrati@univ-ubs.fr)

| **Footprint ID** | **Laterality** | **3D** | **Footprint length (cm)** | **Orientation (°)** | **Estimated stature (cm)** | | | **Estimated age class** |
| --- | --- | --- | --- | --- | --- | --- | --- | --- |
|  |  |  |  |  | **Dingwall et al., 2013** | **Duveau. 2022** | **Mean** |  |
| C01-01 | right | x | 21.1 | 92.1 | 154.1 | 153.5 | 153.8 | child |
| C01-02 | right | x | 16.6 | 122.2 | 135.7 | 136.4 | 136.0 | child |
| C01-03 | right | x | unknown | 80.4 | not estimated | not estimated | not estimated | not estimated |
| C01-04 | unknown | x | 24.9 | 102.1 | 169.7 | 167.9 | 168.8 | adolescent |
| C01-05 | left | x | 18.5 | 135.6 | 143.5 | 143.6 | 143.5 | child |
| C01-06 | left | x | 18.5 | 42.5 | 143.5 | 143.6 | 143.5 | child |
| C01-07 | right | x | 26.5 | 178.6 | 176.3 | 174.0 | 175.1 | adult |
| C01-08 | unknown | x | 23.4 | 248.1 | 163.5 | 162.2 | 162.9 | adolescent |
| C01-09 | unknown | x | 22.5 | 281.2 | 159.9 | 158.8 | 159.3 | adolescent |
| C01-10 | unknown | x | 28.2 | 263.5 | 183.2 | 180.5 | 181.8 | adult |
| C01-11 | unknown | x | 28.8 | 277.5 | 185.7 | 182.7 | 184.2 | adult |
| C01-12 | right | x | 26.6 | 296.7 | 176.7 | 174.4 | 175.5 | adult |
| C01-13 | unknown |  | unknown | 302.8 | not estimated | not estimated | not estimated | not estimated |
| C01-18 | unknown |  | unknown | 33.9 | not estimated | not estimated | not estimated | not estimated |
| C01-20 | left |  | 24.6 | 98.0 | 168.6 | 166.9 | 167.8 | adolescent |
| C01-25 | right |  | 29.8 | 59.9 | 189.7 | 186.4 | 188.0 | adult |
| C01-26 | left |  | 26.9 | 299.2 | 178.0 | 175.6 | 176.8 | adult |
| C01-28 | right |  | 26.3 | 41.6 | 175.4 | 173.2 | 174.3 | adult |
| C02-01 | right | x | 20.3 | 90.6 | 150.8 | 150.4 | 150.6 | child |
| C02-04 | unknown |  | 19.9 | 42.1 | 149.2 | 148.9 | 149.0 | child |
| C02-05 | unknown |  | 21.9 | 316.6 | 157.3 | 156.4 | 156.8 | adolescent |
| C02-07 | right |  | 24.7 | 300.8 | 168.7 | 167.0 | 167.8 | adolescent |
| C02-08 | right |  | 20.8 | 294.0 | 153.0 | 152.5 | 152.7 | child |
| C02-09 | unknown |  | 19.4 | 299.4 | 147.1 | 147.0 | 147.1 | child |
| C02-15 | left |  | 21.0 | 283.1 | 153.6 | 153.0 | 153.3 | child |
| C02-18 | right |  | 24.5 | 265.8 | 167.9 | 166.3 | 167.1 | adolescent |
| C03-01 | right | x | 23.9 | 66.7 | 165.6 | 164.1 | 164.9 | adolescent |
| C03-04 | left |  | 13.2 | 138.2 | 121.7 | 123.5 | 122.6 | child |
| C03-05 | right |  | 19.8 | 34.8 | 148.9 | 148.7 | 148.8 | child |
| C03-11 | right |  | 19.1 | 341.8 | 146.0 | 146.0 | 146.0 | child |
| C03-15 | right |  | 22.3 | 281.1 | 158.9 | 157.9 | 158.4 | adolescent |
| C03-16 | left |  | 26.8 | 26.0 | 177.3 | 175.0 | 176.2 | adult |
| C03-21 | left | x | 16.2 | 72.0 | 134.0 | 134.9 | 134.4 | child |
| C04-01 | right |  | 26.1 | 85.8 | 174.6 | 172.5 | 173.5 | adult |
| C04-02 | right | x | 21.8 | 129.9 | 157.0 | 156.1 | 156.6 | adolescent |
| C04-04 | left | x | 19.2 | 275.5 | 146.3 | 146.3 | 146.3 | child |
| C04-07 | left | x | 22.8 | 319.8 | 161.1 | 159.9 | 160.5 | adolescent |
| C04-08 | left |  | 24.8 | 68.6 | 169.1 | 167.4 | 168.3 | adolescent |
| C04-12 | left |  | 22.9 | 109.6 | 161.6 | 160.4 | 161.0 | adolescent |
| C04-17 | right |  | 28.0 | 268.0 | 182.5 | 179.8 | 181.2 | adult |
| C04-21 | left |  | 21.2 | 226.8 | 154.7 | 154.0 | 154.3 | child |
| C04-24 | left |  | 25.5 | 217.7 | 172.3 | 170.3 | 171.3 | adult |
| C04-25 | left |  | 21.7 | 271.0 | 156.6 | 155.8 | 156.2 | adolescent |
| C04-27 | right |  | 23.9 | 237.0 | 165.5 | 164.0 | 164.7 | adolescent |
| C05-01 | left | x | 28.0 | 217.0 | 182.4 | 179.7 | 181.1 | adult |
| C05-02 | unknown | x | 24.6 | 17.9 | 168.5 | 166.8 | 167.6 | adolescent |
| C05-05 | right | x | 26.7 | 42.7 | 177.1 | 174.8 | 175.9 | adult |
| C05-16 | right | x | 30.0 | 59.2 | 190.6 | 187.3 | 189.0 | adult |
| C05-21 | unknown |  | 27.6 | 237.5 | 180.8 | 178.2 | 179.5 | adult |
| C05-24 | unknown | x | 17.6 | 115.5 | 139.8 | 140.2 | 140.0 | child |
| C05-25 | left | x | 28.1 | 296.8 | 182.8 | 180.1 | 181.4 | adult |
| C05-26 | left |  | 28.6 | 166.9 | 184.9 | 182.0 | 183.4 | adult |
| C05-28 | right | x | 24.8 | 293.6 | 169.3 | 167.5 | 168.4 | adolescent |
| C05-29 | right | x | 18.2 | 121.3 | 142.2 | 142.5 | 142.3 | child |
| C05-32 | left |  | 25.2 | 22.5 | 170.8 | 168.9 | 169.8 | adolescent |
| C05-36 | right |  | 29.6 | 231.0 | 189.1 | 185.9 | 187.5 | adult |
| C05-37 | right |  | 25.8 | 345.0 | 173.5 | 171.5 | 172.5 | adult |
| C05-39 | right |  | 15.9 | 90.9 | 132.9 | 133.8 | 133.4 | child |
| C05-40 | unknown |  | 20.6 | 238.0 | 152.2 | 151.7 | 152.0 | child |
| C05-43 | left |  | 28.8 | 340.0 | 185.8 | 182.9 | 184.3 | adult |
| C05-46 | right |  | 21.7 | 3.6 | 156.6 | 155.8 | 156.2 | adolescent |
| Ca-02 | left | x | 22.8 | 138.0 | 161.1 | 159.9 | 160.5 | adolescent |
| Ca-03 | right | x | 25.1 | 165.8 | 170.5 | 168.7 | 169.6 | adolescent |
| OC001 | right |  | 18.6 | 356.5 | 143.7 | 143.9 | 143.8 | child |
| OC003 | left |  | 18.3 | 300.7 | 142.6 | 142.8 | 142.7 | child |
| OC005 | right |  | 23.6 | 110.3 | 164.4 | 163.0 | 163.7 | adolescent |
| OC006 | right |  | 22.6 | 351.0 | 160.4 | 159.3 | 159.9 | adolescent |
| OC010 | left |  | unknown | 7.5 | not estimated | not estimated | not estimated | not estimated |
| OC014 | right |  | 24.2 | 46.2 | 166.8 | 165.2 | 166.0 | adolescent |
| OC017 | left |  | 26.2 | 128.2 | 174.9 | 172.7 | 173.8 | adult |
| OC022 | right |  | 12.7 | 119.8 | 119.8 | 121.7 | 120.8 | child |
| OC023 | right |  | 14.0 | 324.0 | 124.8 | 126.4 | 125.6 | child |
| OC025 | right |  | 21.1 | 220.9 | 154.0 | 153.4 | 153.7 | child |
| OC026 | right |  | 26.6 | 167.7 | 176.8 | 174.5 | 175.7 | adult |
| OC028 | right |  | 19.0 | 152.2 | 145.4 | 145.4 | 145.4 | child |
| OC029 | left |  | 18.6 | 140.6 | 144.0 | 144.1 | 144.0 | child |
| OC033 | right |  | 28.8 | 273.2 | 185.8 | 182.9 | 184.3 | adult |
| OC042 | unknown |  | 17.8 | 281.9 | 140.6 | 140.9 | 140.8 | child |
| OC046 | left |  | 23.1 | 306.9 | 162.4 | 161.2 | 161.8 | adolescent |
| OC048 | right |  | 17.9 | 153.5 | 141.1 | 141.4 | 141.3 | child |
| OC052 | left |  | 29.9 | 275.2 | 190.2 | 186.9 | 188.5 | adult |
| OC062 | unknown |  | 14.1 | 247.3 | 125.6 | 127.0 | 126.3 | child |
| OC063 | left |  | 18.8 | 106.8 | 144.5 | 144.6 | 144.6 | child |
| OC064 | left |  | 14.0 | 20.1 | 124.9 | 126.4 | 125.6 | child |
| OC066 | left |  | 21.6 | 112.0 | 156.3 | 155.5 | 155.9 | adolescent |

**Table S1.** Inventory of the footprints discovered at Larache site. Statures estimated from the total length of the footprints using the average of the estimates obtained from: 1) 15% ratio between foot length and stature. 2) the relationship used by Dingwall and his collaborators. and 3) that used by Duveau and his collaborators.


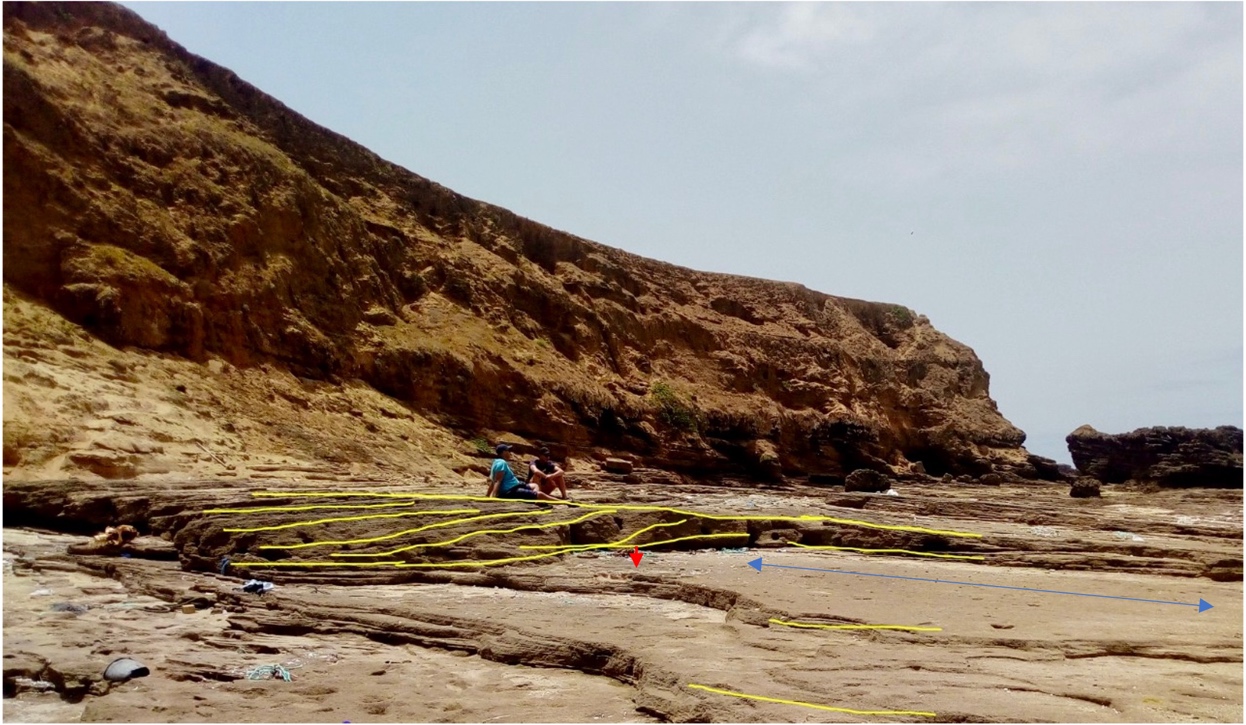


**Figure S1.** Side view of the tracked site. The yellow lines indicate crossbedded sands that constitute the sand bar. Note the seaward-inclined slope of the bar. The lower yellow line corresponds to the lower tracked surface where the sediment sample was collected for OSL dating (red arrow). The coordinates of the sampling point are: (X: 428456.313 - Y: 506198.258 – Z: 1.7). The blue double-pointed arrow corresponds to the area where two cross trackways were found (see Fig.2b).


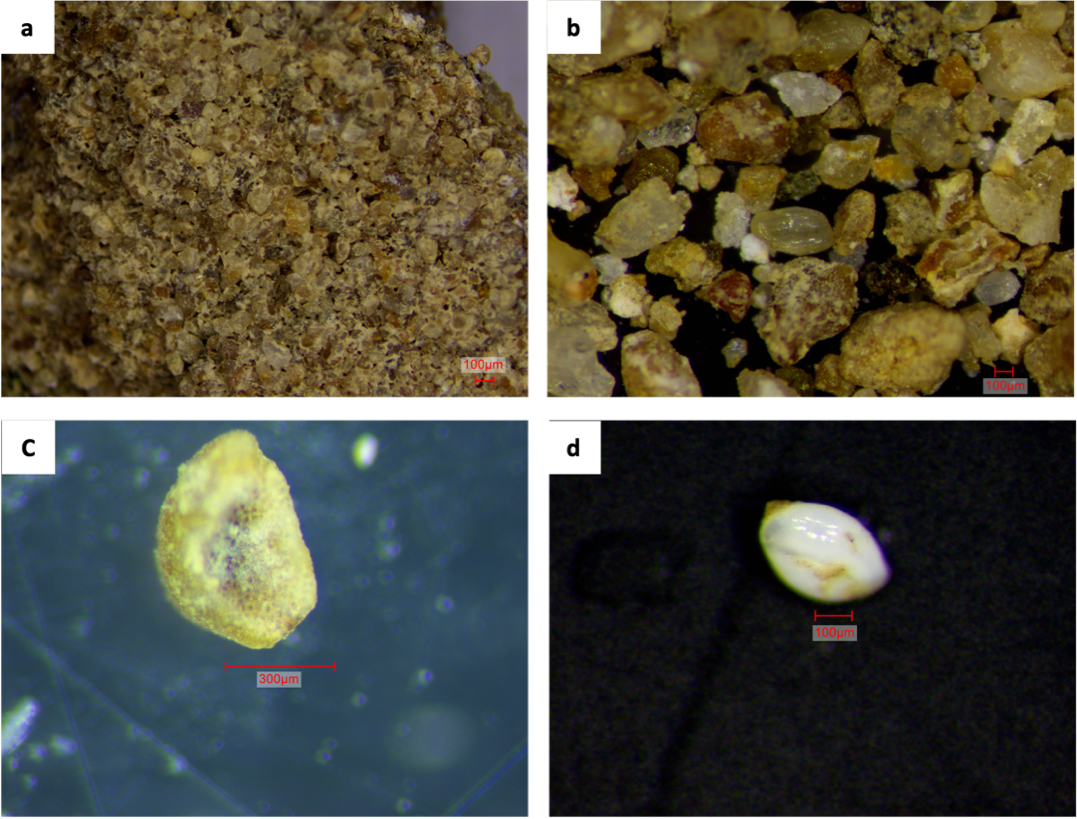


**Figure S2.** Sediment analysis. (a) Plane-polarised light petrographic microscope image of the sandstone sample used for OSL datation showing *quartz grains of medium-fine sand*. (b) Cross-polarised light petrophotographic microscope image of the sandstone sample used for OSL datation *quartz grains of medium-fine sand*. (c) Image of isolated *echinoderm*. (d) Image of isolated *benthic foraminifera (Quinqueloculina sp.).* The sample was observed by a ZEISS Discovry B8 Microscope and the images were taken by Deltapix camera (Invenio 12EIII).


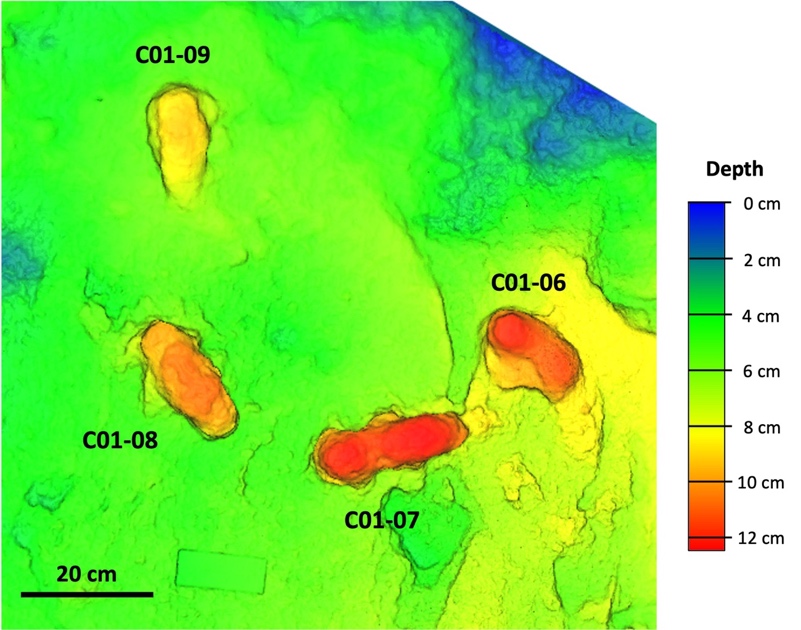

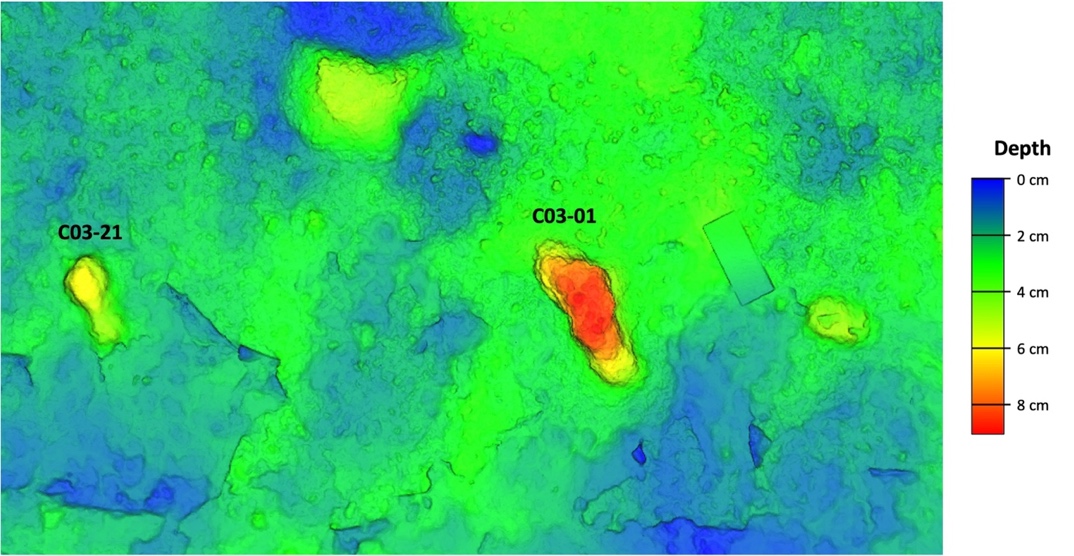


**Figure S3.** Depth map of a group of Larache footprints.


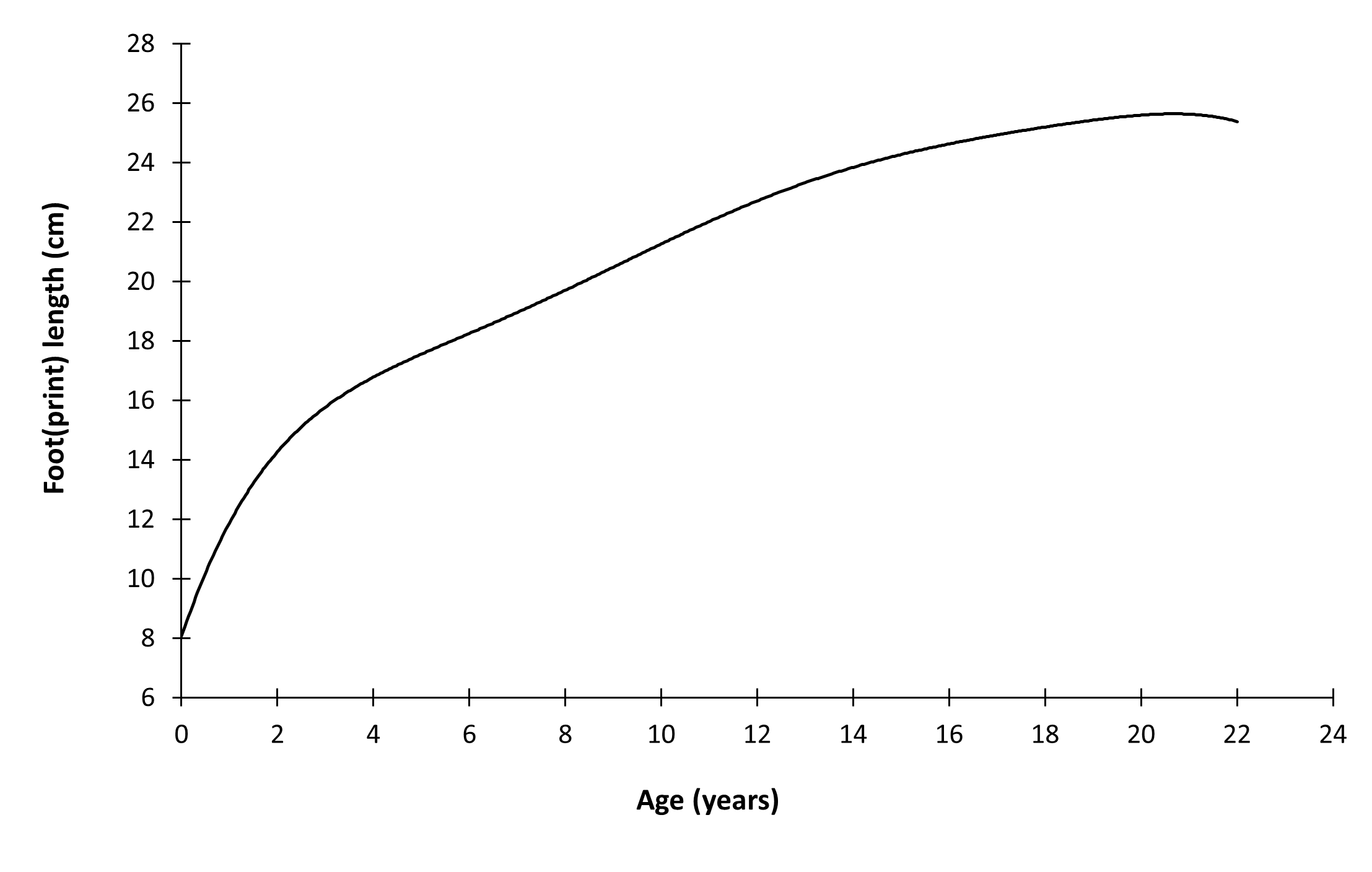


**Figure S4.** Average growth curve of foot length obtained for different modern populations^69.70.71.72.73.74^ (Total: 12.000 individuals).


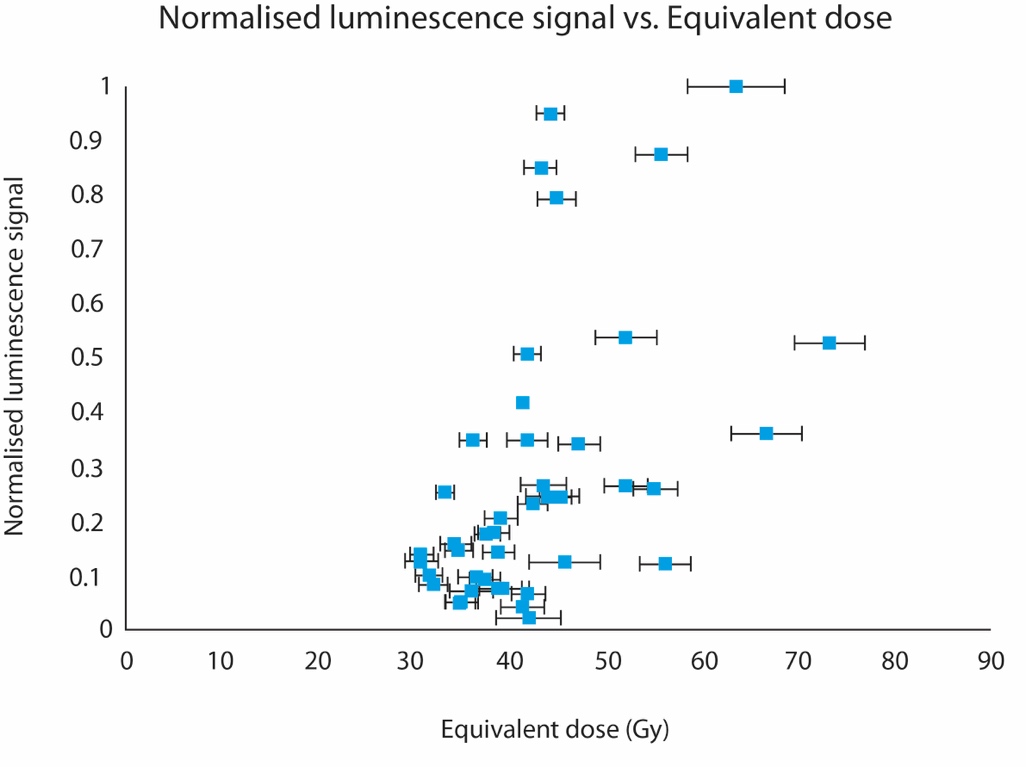


**Figure S5**. Dose distribution obtained from the OSL measurements. Normalized OSL signal is plotted as a function of the individual equivalent dose values.


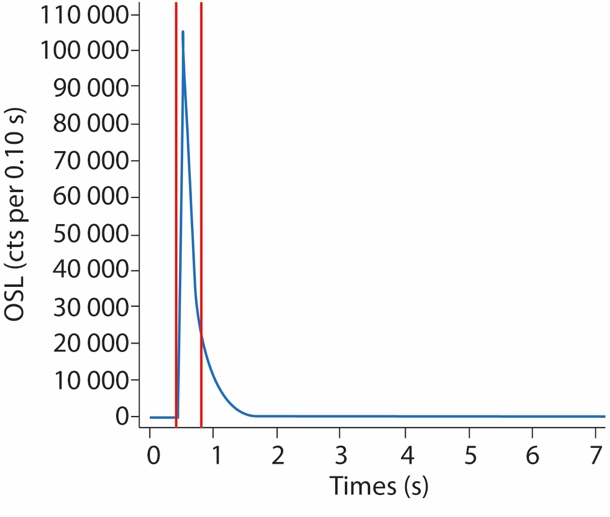

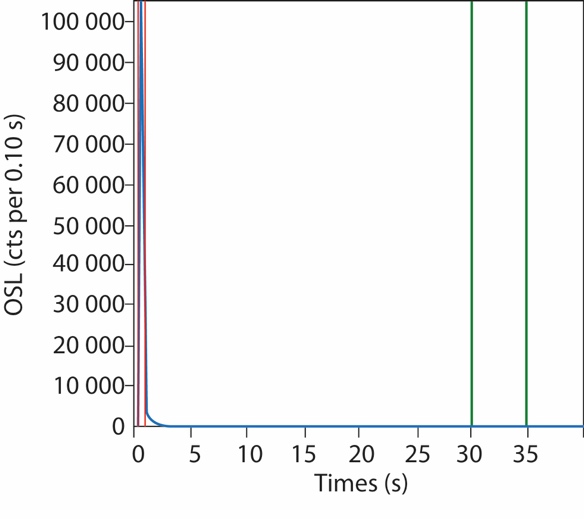


**Figure S6**. Example of the OSL decay curves obtained. OSL signal is shown in blue. Red and green lines represent the integration limits selected for the curve and the background. respectively (right). Fast component section of the OSL curve (left).


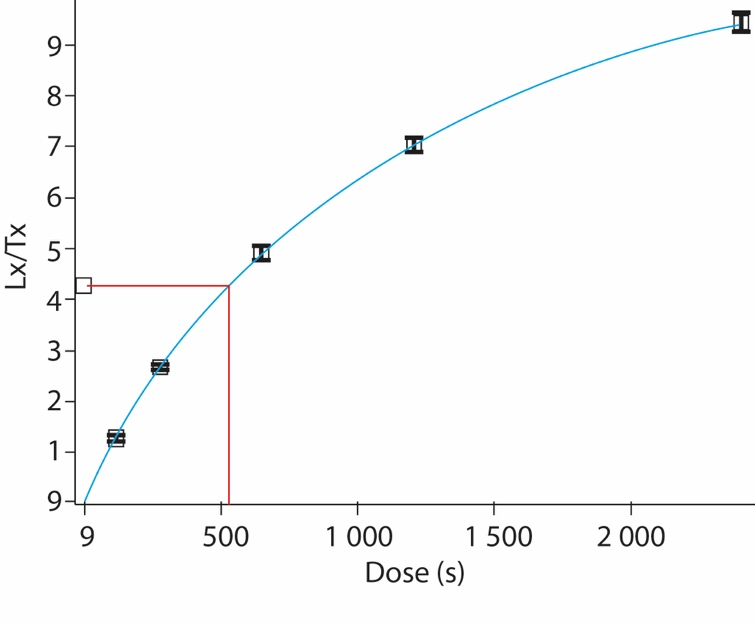


**Figure S7**. Example of the dose response curves obtained.


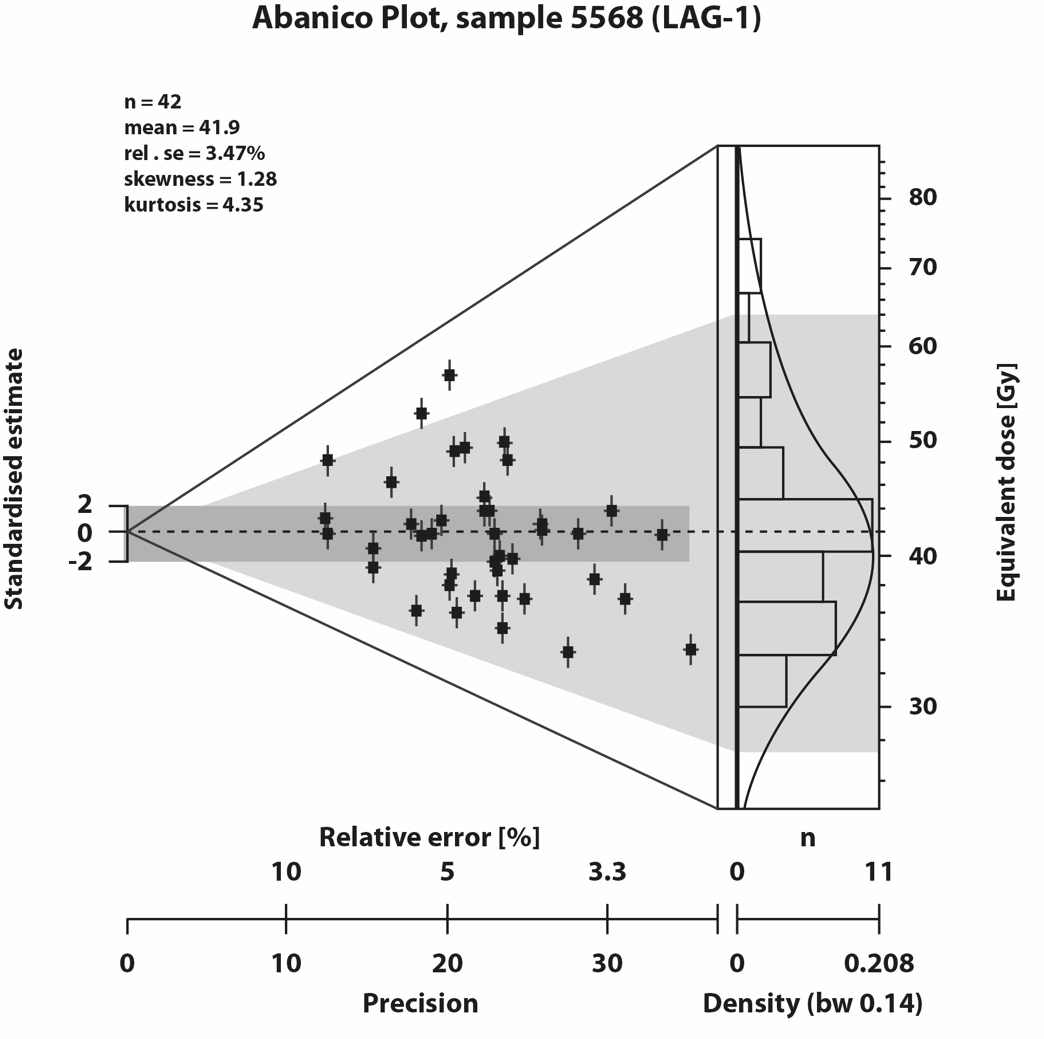


**Figure S8**. Abanico plot for the OSL measurements.
